# Discovery of a tyrosine-rich sporocyst wall protein in *Eimeria tenella*

**DOI:** 10.1186/s13071-016-1410-z

**Published:** 2016-03-02

**Authors:** Robert A. Walker, Alisson Niepceron, Chandra Ramakrishnan, Laura Sedano, Adrian B. Hehl, Fabien Brossier, Nicholas C. Smith

**Affiliations:** Queensland Tropical Health Alliance Research Laboratory, Australian Institute of Tropical Health and Medicine, James Cook University, Cairns Campus, McGregor Road, Smithfield, QLD 4878 Australia; Institute of Parasitology, University of Zurich, Winterthurerstrasse 266a, CH-8057 Zurich, Switzerland; INRA, UMR1282, Infectiologie et Santé Publique, Laboratoire Apicomplexes et Immunité Mucosale, F-37380 Nouzilly, France; Université François-Rabelais de Tours, UMR1282 Infectiologie et Santé Publique, F-37000 Tours, France

**Keywords:** *Eimeria*, Coccidia, Apicomplexa, Oocyst, Sporocyst, Dityrosine bond

## Abstract

**Background:**

*Eimeria *is an important genus of apicomplexan parasites. A defining feature of these parasites is the oocyst, which is transmitted into the environment *via* the faeces of definitive hosts. The oocyst wall contains cross-linked, tyrosine-rich proteins and protects eight infectious sporozoites, housed in pairs within a second walled structure, the sporocyst. The biochemical basis for sporocyst wall formation is not known.

**Findings:**

Here, we report the discovery of a novel tyrosine-rich protein, *Et*SWP1, in *Eimeria tenella*. Like the tyrosine-rich proteins of the oocyst wall, *Et*SWP1 is an intrinsically disordered protein with the tyrosine residues concentrated in a specific region of the protein, located immediately following the region of intrinsic disorder. We engineered *E. tenella* to express mCherry-tagged *Et*SWP1 and showed that the tagged protein localises specifically to sporocyst walls*,* indicating that the biochemistry of sporocyst wall assembly is analagous to that of oocyst walls.

**Conclusions:**

Tyrosine-rich proteins are known to be key components of the oocyst wall and we now demonstrate, using gene and protein analyses combined with genetic manipulation, that a novel tyrosine-rich protein is specific for the sporocyst wall. This finding is important because it shows that the biochemistry of these two distinct walls is similar and, hence, brings targeted disruption of sporulation and, therefore, potential neutralisation of oocysts in the environment, a step closer.

**Electronic supplementary material:**

The online version of this article (doi:10.1186/s13071-016-1410-z) contains supplementary material, which is available to authorized users.

## Findings

Apicomplexan parasites of the genus, *Eimeria,* cause coccidiosis in a variety of livestock and poultry; in the case of the latter, the industry loses in excess of US$2 billion dollars per year due to coccidiosis [1]. These obligate intracellular parasites are highly contagious, due primarily to the resilient nature of the oocyst, an important developmental stage that is transmitted into the environment *via* the faeces of definitive hosts. The oocyst wall is the primary barrier between the harsh external environment and the infectious cargo, eight dormant sporozoites. However, these sporozoites are further housed in pairs within four sporocysts, a structure that is itself characterised by a protective barrier, the sporocyst wall.

Several oocyst wall proteins have been identified and characterised to different degrees [2] including: a cysteine-rich protein, *Et*OWP6, which is found in the outer layer of the oocyst wall and is related to a family of proteins found in other apicomplexan parasites, namely *Cryptosporidium parvum* and *Toxoplasma gondii;* a histidine-rich protein, which localises to the inner oocyst wall; and, GAM56 and GAM82, proteins that are found in wall forming bodies type 2 of macrogametocytes, contain tyrosine-rich regions, and are processed into smaller, tyrosine-rich proteins, which are incorporated into the inner layer of the oocyst wall. GAM56 and GAM82 are also the major components of a transmission blocking vaccine to prevent coccidiosis in poultry [3]. In contrast to oocysts, little is known about the biochemical composition of the sporocyst wall of coccidian parasites [4] despite recent transcriptome profiling of different *E. tenella* developmental stages [5, 6].

**Fig. 1:**
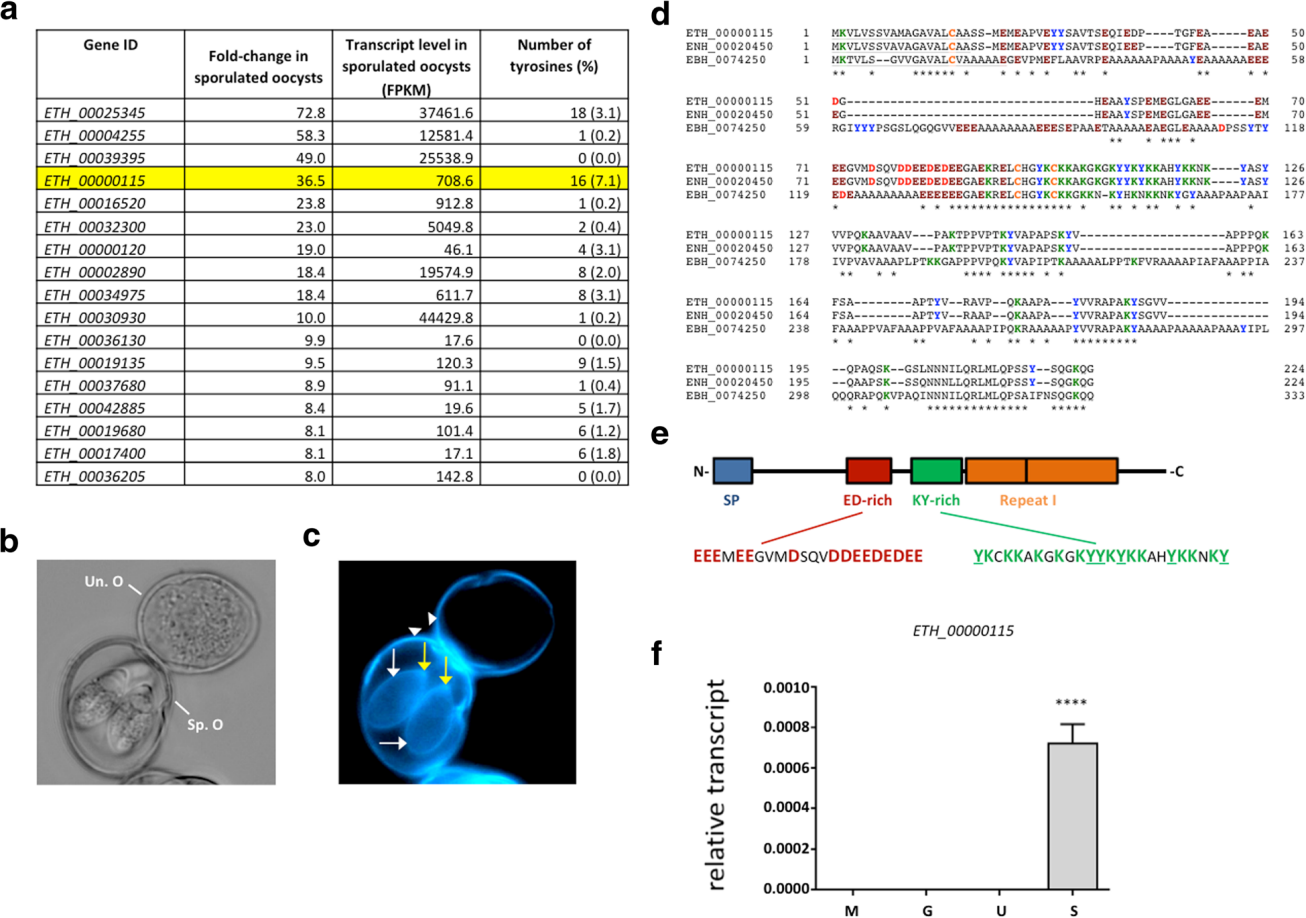
Identification of a tyrosine-rich protein transcribed specifically in sporulated oocysts of *Eimeria tenella.* **a** An integrative search for *E. tenella* genes with increased expression during sporulation was carried out using the New Search option available at www.toxodb.org, following the path New Search > Search for Genes > Transcript Expression > RNA Seq Evidence. The data set “Life Cycle Stage Transcriptomes (Reid)” was selected for *E. tenella* strain Houghton, using the FC (fold-change) option. The search identified 17 protein-coding genes that are upregulated with a fold change of ≥8 in the sporulated oocyst sample compared with the maximum expression value recorded for any of the reference samples, including unsporulated oocyst, sporozoite and 2^nd^ generation merozoite. **b** Brightfield and **c** autofluorescence (UV excitation wavelength = 385 nm) microscopy of a bleached unsporulated (Un. O; top right) and a bleached sporulated (Sp. O; bottom left) oocyst of *E. tenella* indicates the possible presence of dityrosine bonds in the inner wall (bleaching removes the outer wall) of both the unsporulated and sporulated oocyst (white arrow-heads) and in the sporocyst walls (white arrows), including the stieda bodies (yellow arrows)*.* Microscopy was done on a Zeiss Axiovert 200 microscope equipped with the Apotome imaging system. Images were generated and analysed using the Axiovision Software (Carl Zeiss SA). **d** The identification of potential ETH_00000115 (NCBI Reference Sequence: XP_013233236.1) homologues was carried out using BLASTP on the non-redundant NCBI database or on www.toxodb.org and alignments generated using the CLUSTAL O (1.2.1) multiple sequence alignment tool at http://www.ebi.ac.uk/Tools/msa/clustalo/. Conventional BLASTP analysis revealed only a single, highly conserved protein (96 % identity with ETH_00000115) in *Eimeria necatrix* (ENH_00020450, NCBI Reference Sequence: XP_013439901.1); however, searching for the KY-rich sequence, YKCKKAKGKGKYYKK, uncovered a further putative homologue in *Eimeria brunetti* (EBH_0074250, GenBank Reference Sequence: CDJ54052.1; 48 % identity with ETH_00000115), the ED-rich region and extended C-terminal of which are interspersed with several poly-alanine (A) sequences. The putative signal peptides for the three proteins are underlined. Residues of aspartic acid are highlighted by , glutamic acid by , lysine by , tyrosine by  and cysteine by . Amino acid residues that are conserved across all three species are indicated by *. **e** A graphic depiction of the predicted ETH_00000115 protein highlighting a lysine (K) and tyrosine (Y)-rich region flanked by a negatively-charged, aspartic acid (D) and glutamic acid (E)-rich region, and a weak repeat sequence. **f** Quantitative reverse-transcriptase PCR carried out on different developmental stages of *E. tenella* [6] confirms the sporulated oocyst-specific expression of *ETH_00000115.* The relative transcript abundance of *ETH-00000115* was determined relative to the *et18s* small subunit ribosomal RNA for each developmental stage (M = merozoites, G = gametocytes, U = unsporulated oocysts, S = sporulated oocysts). **** Indicates a statistically significant difference for sporulated oocysts vs every other stage at *P* < 0.001 (*n* = 3 samples per developmental stage; one-way ANOVA and Bonferroni multiple comparison post-hoc tests using GraphPad Prism® Version 6.03, GraphPad Software Inc., USA)

In order to identify a sporocyst wall-specific protein, we mined the *E. tenella* genome database using relevant transcript expression data [7] available at www.toxodb.org. Since the sporocyst wall is synthesised only during sporulation, we worked on the assumption that the expression of a sporocyst wall protein would be upregulated specifically in sporulated oocysts. A search for protein coding genes with at least 8-fold increased transcript levels in sporulated oocysts compared to any other stage available (including 2^nd^ generation merozoites, sporozoites and unsporulated oocysts) yielded a total of 17 genes (Fig. 1a). With the notable exception of the sporozoite surface antigen, *Et*SAG10 (encoded by *ETH_00034975*), the proteins encoded by these transcripts have no known function or annotation. Expression of *ETH_00034975* is known to be upregulated dramatically in the second half of sporulation and maintained in sporozoites and merozoites, indicative of its function as a surface protein on invasive stages of *E. tenella* [8].

Amongst the 17 genes listed in Fig. 1a, *ETH_00000115* stands out; the gene model is predicted to encode a protein that has 7.1 % tyrosine residues (16 of 224 amino acids), which is more than twice that of the next highest (encoded by *ETH_00025345*). This is potentially significant because, like the oocyst wall, the sporocyst wall of *E. tenella* fluoresces blue under UV excitation (Fig. 1b,c), a feature that indicates the possible presence of cross-linked tyrosine-rich proteins [9]. Moreover, the predicted amino acid sequence (Fig. 1d) of ETH_00000115 shows that most of these tyrosines are confined to a specific region of the protein, which is analogous to the amino acid sequences of GAM56 and GAM82.

We searched for homologues of ETH_00000115 in other Coccidia but, with the exception of a highly conserved protein in *Eimeria necatrix* (Fig. 1d), conventional BLASTP analysis against non-redundant protein databases failed to identify homologues of ETH_00000115, including in any other Apicomplexa. Furthermore, ETH_00000115 showed no homology to any previously described protein domains, except for a predicted N-terminal signal peptide, identified using the SignalP 4.1 Server at www.cbs.dtu.dk/services/SignalP [10]. However, by searching for the KY-rich sequence, YKCKKAKGKGKYYKK, we were able to identify a putative homologue in *Eimeria brunetti,* the otherwise ED-rich region and C-terminal sequence of which are dominated by numerous poly-alanine stretches (Fig. 1d). Like ETH_00000115, the *E. necatrix* and *E. brunetti* proteins contain a distinct KY-rich region. All three proteins contain only three cysteine residues but these appear to be strategically placed, with the first near the cleavage site of the signal peptides and the other two at the start of the KY-rich regions, separated by a conserved HGYK sequence.

In both GAM56 and GAM82, the tyrosine-rich regions are also rich in serine residues whereas in ETH_00000115, the tyrosine residues are accompanied by lysine residues. Upstream of, and almost directly adjacent to the lysine and tyrosine-rich (or KY-rich) domain is a 22-residue acidic domain or “ED-rich” domain, which is composed of ten glutamic acid (E) and five aspartic acid (D) residues. Directly downstream of the KY-rich domain are two copies of an imperfect repeat, we have called Repeat 1 (Fig. 1e).

Quantitative reverse-transcriptase PCR (qRT-PCR) was used to assess stage-specific expression of *ETH_00000115.* The relative transcript abundance of *ETH-00000115* was determined relative to the *et18s* small subunit ribosomal RNA for each developmental stage using chicken infection regimens and PCR protocols exactly as described previously [6]. The forward primer was 5′-CGCTGAGGAAGAAATGGAAG-3′ and the reverse primer was 5′-TAAGTGCAAAAAGGCCAAGG-3′. Analysis by qRT-PCR confirms that *ETH_00000115* transcript is highly abundant in sporulated oocysts but essentially absent in other stages, such as merozoites, gametocytes and unsporulated oocysts (Fig. 1f).

**Fig. 2:**
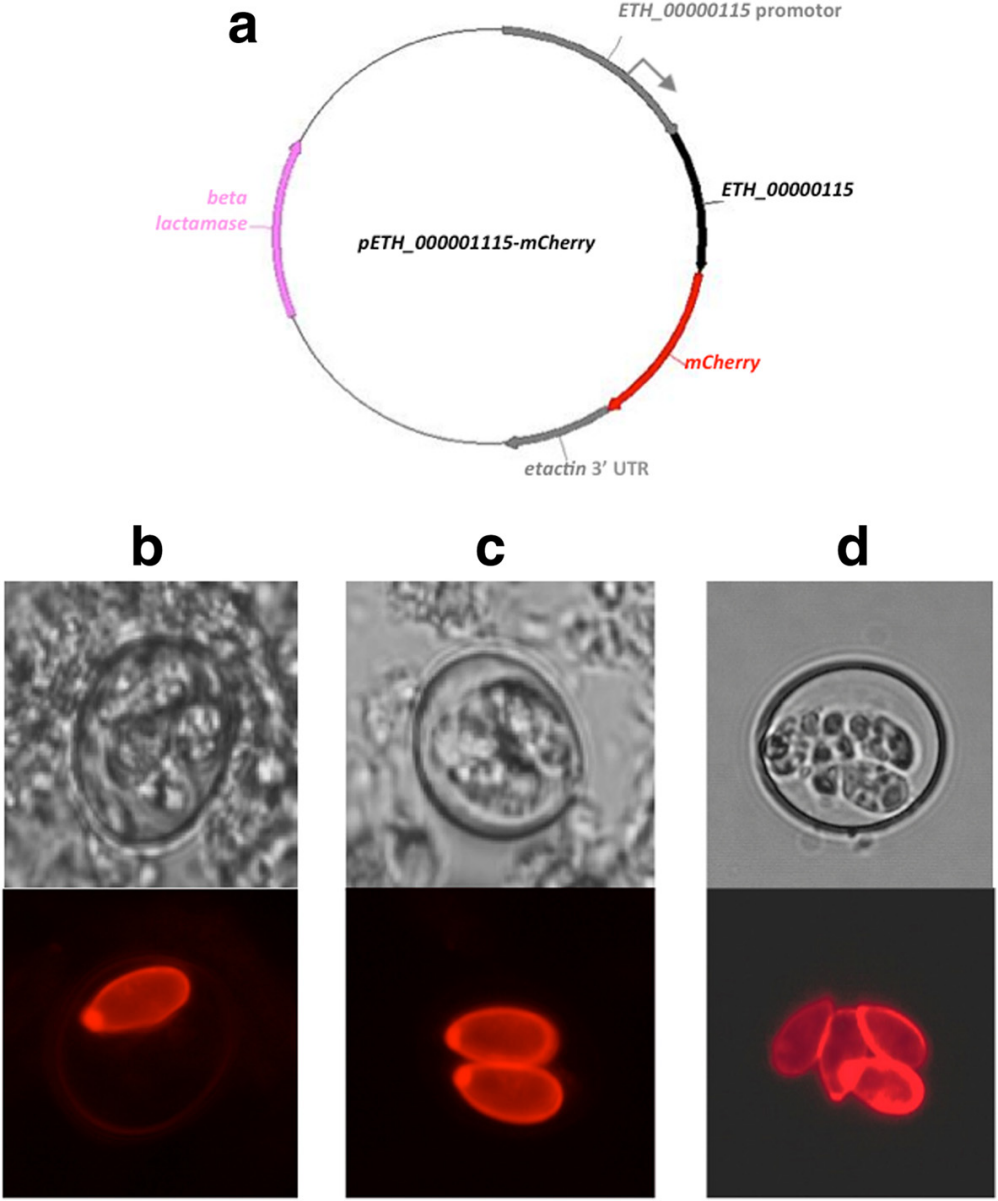
The tyrosine-rich protein encoded by *ETH_00000115* is a sporocyst wall specific protein in *Eimeria tenella*. **a** In order to determine the localisation of the tyrosine-rich protein encoded by *ETH_00000115* within sporulated oocysts*,* a reporter plasmid p*ETH_00000115-mCherry* was engineered using the mCherry core construct-1 as a parental plasmid. As there is no predicted intron for *ETH_00000115*, the putative promoter (982 bp upstream of the predicted start codon) and coding sequence of *ETH_00000115* could be PCR amplified from *E. tenella* genomic DNA as one contiguous product (136,055 to 134,402 from supercontig HG675721) using the forward primer, MluI-*ETH_00000115*_F (5'-GGGGATTTTTTGGGATGG-3'), and the reverse primer, SalI-*ETH_00000115*_R (5'-GCAGGGCAAGCAAGGC-3'). The PCR product was cloned into the mCherry core construct-1 via MluI and SalI, replacing the *etmic1* promoter and allowing read-through from the *ETH_00000115* coding sequence to the *mCherry* coding sequence. Amplification of DNA for cloning was carried out by polymerase chain reaction using Pfu DNA Polymerase (Thermo Scientific) according to the manufacturer’s instructions. Transfection of *E. tenella* sporozoites was carried out as described previously [11]. **b**, **c**, **d** Brightfield (upper panels) and red fluorescence (wavelength = 590 nm, lower panels) microscopy of p*ETH_00000115-mCherry* sporulated oocysts of *E. tenella* confirms that the tyrosine-rich protein encoded by *ETH_00000115* is expressed specifically in the sporocyst wall. Recombination resulting from pairing of transfected and non-transfected gametes means that one, two or four sporocysts within any single oocyst may display the mCherry signal; **b** shows a single sporocyst of four, **c** shows two sporocysts of four and **d** shows all four sporocysts exhibiting fluorescence. Microscopy was done on a Zeiss Axiovert 200 microscope equipped with the Apotome imaging system. Images were generated and analysed using the Axiovision Software (Carl Zeiss SA)

To test the hypothesis that ETH_00000115 is a component of the sporocyst wall, we engineered *E. tenella* to express an mCherry-tagged version of ETH_00000115. We chose this approach over conventional immunofluorescence to avoid any complications with antibody cross-reactivity to other tyrosine-rich proteins present in *E. tenella.* A reporter construct, p*ETH_00000115-mCherry*, was engineered for transfection in *E. tenella* (Fig. 2a). The reporter plasmid contains the complete coding sequence of *ETH_00000115* under the control of its own putative promoter (*i.e.*, within ~1 kb of sequence upstream of the predicted start codon), in frame with the coding sequence of the fluorescent protein, mCherry, followed by the 3′ UTR of *etactin*. Freshly excysted *E. tenella* sporozoites were transfected with p*ETH_00000115-mCherry*, re-inoculated into naive chickens and oocysts purified 7 days later, as described previously [11]. Microscopic examination of fluorescent oocysts revealed mCherry fluorescence localising specifically to sporocyst walls (Fig. 2b, c). We did not detect mCherry fluorescence in enteric parasite stages such as merozoites and gametocytes. This confirms that ETH_00000115 is a *bona fide* sporocyst wall protein, which we have therefore named and, from hereon, refer to as “*Et*SWP1” for ***E****imeria ****t****enella ***S**porocyst **W**all **P**rotein 1. We observed that mCherry + oocysts could have one, two or four mCherry + sporocyst walls, a result explained by the relatively low efficiency of transfection and the consequent pairing of genetically different gametes, underscored by the high recombination frequency observed during meiosis in *Eimeria* species [12].

The tyrosine-rich oocyst wall protein, GAM56 is known to possess significant regions of intrinsic disorder within its structure, which is dominated by random coils, with some helices but few sheets/strands [13]. Two repercussions of this are predicted [13]: first, intrinsically disordered proteins are susceptible to proteolytic cleavage, as observed for GAM56; and, second, the inherent flexibility of disordered proteins may increase the possibility of tyrosine residues from individual tyrosine-rich proteins coming into the proximity of each other more readily, thereby facilitating crosslinking. We used DISOPRED3 [14] to analyse the amino acid sequences of *Et*SWP1, *En*SWP1 and *Eb*SWP1 to determine if these proteins possess similar secondary structures to GAM56; DISOPRED3 has been used recently, with significant success, to identify regions of intrinsic disorder in the structure of apicomplexan proteins [15]. We found that all three SWP1s were dominated by coil structures, with some helices and very little sheet/strand structures (Additional file 1: Figure S1). Analyses using I-Tasser [16–19] generated similar predictions (Additional file 2: Figure S2). DISOPRED3 also predicted significant regions of intrinsic disorder within all the SWP1s, most notably in the ED-rich region preceding the KY-rich sequence (Additional file 3: Figure S3); GAM56 is, likewise, intrinsically disordered just prior to the tyrosine-rich region. We therefore predict that SWP1 behaves similarly to GAM56 in wall assembly.

BLASTP searches also failed to identify any homologues of *Et*SWP1 in the cyst forming coccidians, *Toxoplasma gondii, Neospora caninum* or *Hammondia hammondi,* but, by searching for proteins with similar percentages of lysine, tyrosine, glutamic acid and aspartic acid, three sequences (TGME49_037080, NCLIV_050960 and HHA_237080) that are highly conserved across these three parasites were discovered (each with ~21 % identity with *Et*SWP1). However, TGME49_037080 has already been identified as more likely to be a component of the oocyst wall and not the sporocyst wall of *T. gondii* by transcriptomic [20] and proteomic [21] analyses.

There are other tyrosine-rich proteins indicated by transcriptomic analysis of *T. gondii* oocysts as being potential components of the sporocyst wall [20] but the localisation of these proteins specifically to the sporocyst wall has not been confirmed. The discovery that *Et*SWP1 is targeted specifically to the sporocyst wall is important because, in combination with the recent finding that the sporocyst wall contains cysteine-rich proteins [22], it shows that the construction of the sporocyst wall is biochemically similar to the oocyst wall despite being assembled *via* a different cell biology mechanism; in contrast to oocyst wall formation, which occurs *via* sequential, coordinated migration and disaggregation of veil forming bodies, wall forming bodies type 1 and wall forming bodies type 2 [23], the wall of the sporocyst forms *via* condensation of cytoplasmic material not contained in defined organelles or vesicles [22, 24]. Our discovery of a tyrosine-rich protein in the sporocyst wall brings targeted disruption of sporulation and, therefore, potential neutralisation of oocysts in the poultry house, a step closer.

## Ethics statement

Animal experimentation was conducted under protocols approved by the University of Technology, Sydney Animal Ethics Committee (protocol numbers 2008–96 and 2008–188) under a protocol (registration number 2012-11-9) and/or by the ethics committee, CEEA VdL, and according to French legislation (French Government Decree 2001–464) and EEC regulations (86/609/CEE).

## Additional files

Additional file 1: Figure S1. Secondary structure predictions for SWP1 from (a) *Eimeria tenella,* (b) *Eimeria necatrix* and (c) *Eimeria brunetti,* determined using DISOPRED3 (http://bioinf.cs.ucl.ac.uk/disopred) indicating that all three proteins are dominated by random coils (71%, 71% and 73%, respectively), with significant helices (25%, 25% and 27%, respectively) but few sheet/strand structures (4%, 4% and 0%, respectively). (TIFF 1521 kb) Additional file 2: Figure S2. Secondary structure predictions for SWP1 from (a) *Eimeria tenella,* (b) *Eimeria necatrix* and (c) *Eimeria brunetti,* determined using I-Tasser (http://zhanglab.ccmb.med.umich.edu/I-TASSER/). red H = helix, blue S = strand, C = coil. The lysine/tyrosine-rich region is highlighted in green and the glutamic acid/aspartic acid-rich region in yellow. (TIFF 2702 kb) Additional file 3: Figure S3. Identification of predicted intrinsically disordered structures in SWP1 from (a) *Eimeria tenella,* (b) *Eimeria necatrix* and (c) *Eimeria brunetti,* determined using DISOPRED3 (http://bioinf.cs.ucl.ac.uk/disopred). (TIFF 2702 kb)
