# Supplementary figures and images for: Discovery of a tyrosine-rich sporocyst wall protein in Eimeria tenella
Source: Parasit Vectors. 2016 Mar 2;9:124. doi: 10.1186/s13071-016-1410-z (PMC4776368; doi:10.1186/s13071-016-1410-z)

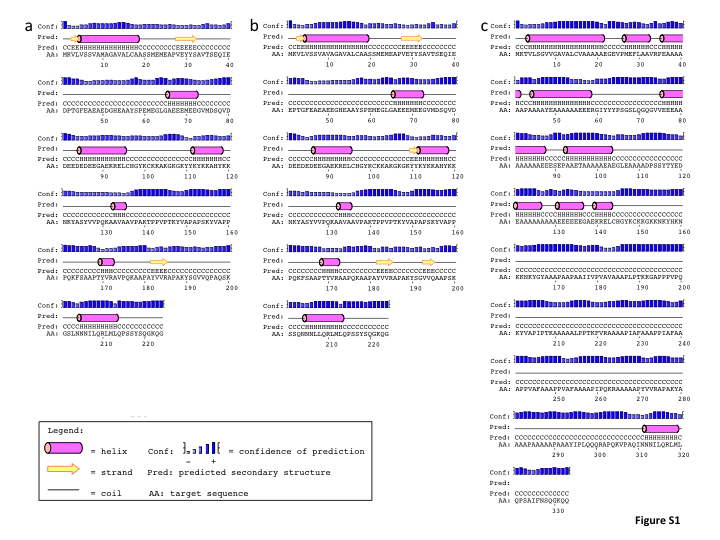

Supplement: Additional file 1: Figure S1. — Secondary structure predictions for SWP1 from (a) Eimeria tenella, (b) Eimeria necatrix and (c) Eimeria brunetti, determined using DISOPRED3 (http://bioinf.cs.ucl.ac.uk/disopred) indicating that all three proteins are dominated by random coils (71%, 71% and 73%, respectively), with significant helices (25%, 25% and 27%, respectively) but few sheet/strand structures (4%, 4% and 0%, respectively). (TIFF 1521 kb) [file 13071_2016_1410_MOESM1_ESM.tiff]

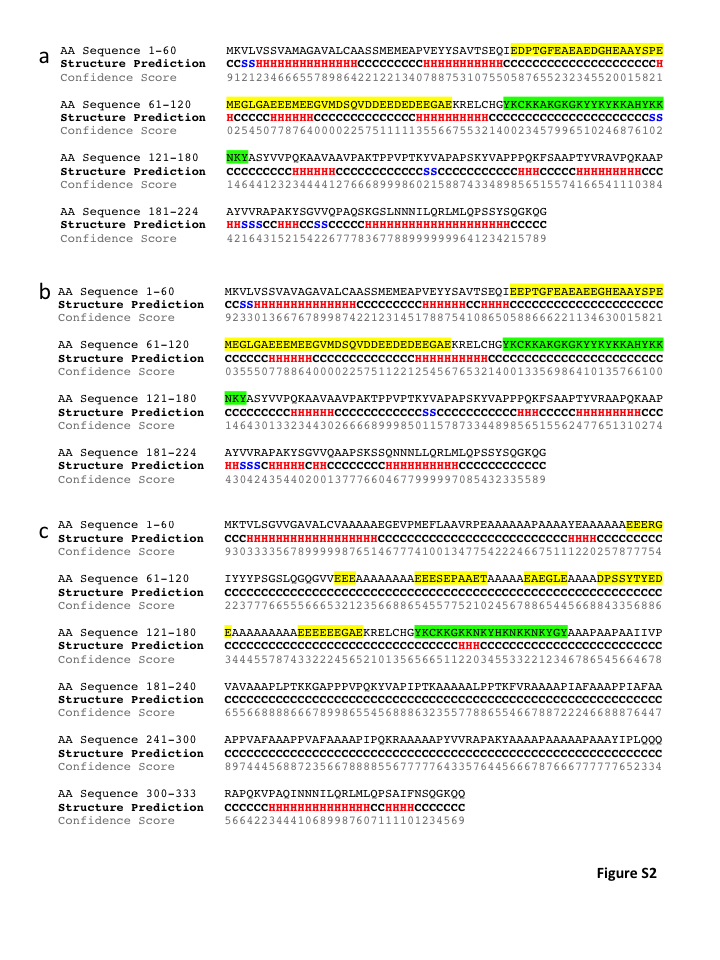

Supplement: Additional file 2: Figure S2. — Secondary structure predictions for SWP1 from (a) Eimeria tenella, (b) Eimeria necatrix and (c) Eimeria brunetti, determined using I-Tasser (http://zhanglab.ccmb.med.umich.edu/I-TASSER/). red H = helix, blue S = strand, C = coil. The lysine/tyrosine-rich region is highlighted in green and the glutamic acid/aspartic acid-rich region in yellow. (TIFF 2702 kb) [file 13071_2016_1410_MOESM2_ESM.tiff]

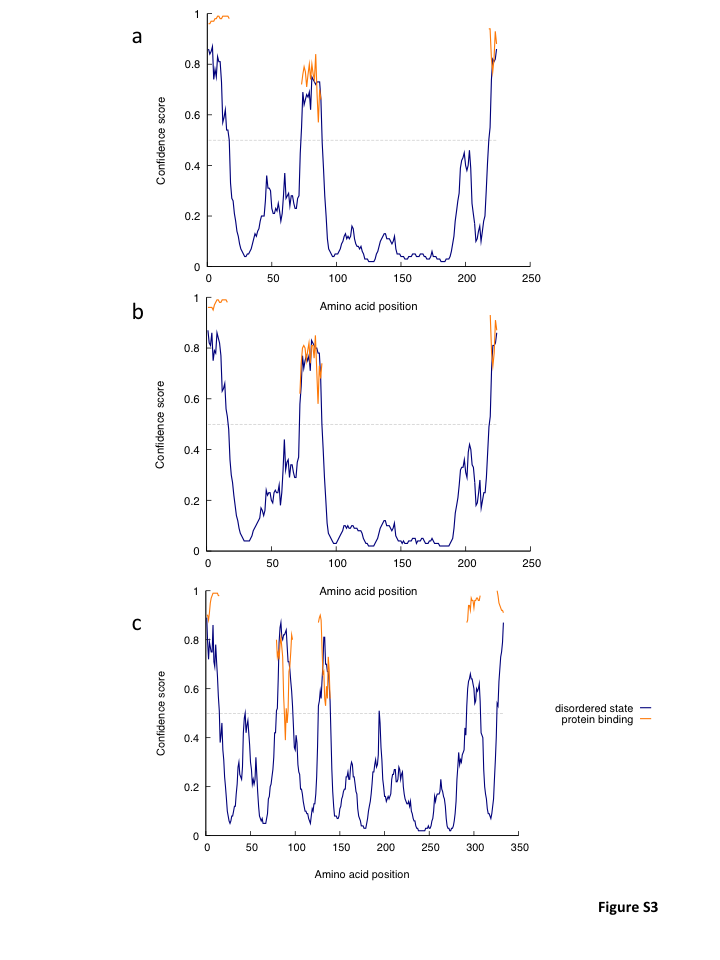

Supplement: Additional file 3: Figure S3. — Identification of predicted intrinsically disordered structures in SWP1 from (a) Eimeria tenella, (b) Eimeria necatrix and (c) Eimeria brunetti, determined using DISOPRED3 (http://bioinf.cs.ucl.ac.uk/disopred). (TIFF 2702 kb) [file 13071_2016_1410_MOESM3_ESM.tiff]
